# System-Wide Profiling by Proteome Integral Solubility Alteration Assay of Drug Residence Times for Target Characterization

**DOI:** 10.1021/acs.analchem.2c03506

**Published:** 2022-11-03

**Authors:** Pierre Sabatier, Christian M. Beusch, Zhaowei Meng, Roman A. Zubarev

**Affiliations:** † Division of Chemistry I, Department of Medical Biochemistry and Biophysics, Karolinska Institutet, Stockholm17177, Sweden; ‡ Department of Surgical Sciences, Uppsala University, Uppsala751 85, Sweden; § Novo Nordisk Foundation Center for Protein Research, University of Copenhagen, Copenhagen2200, Denmark; ∥ Department of Pharmacological & Technological Chemistry, I.M. Sechenov First Moscow State Medical University, Moscow119146, Russia; ⊥ The National Medical Research Center for Endocrinology, Moscow115478, Russia

## Abstract

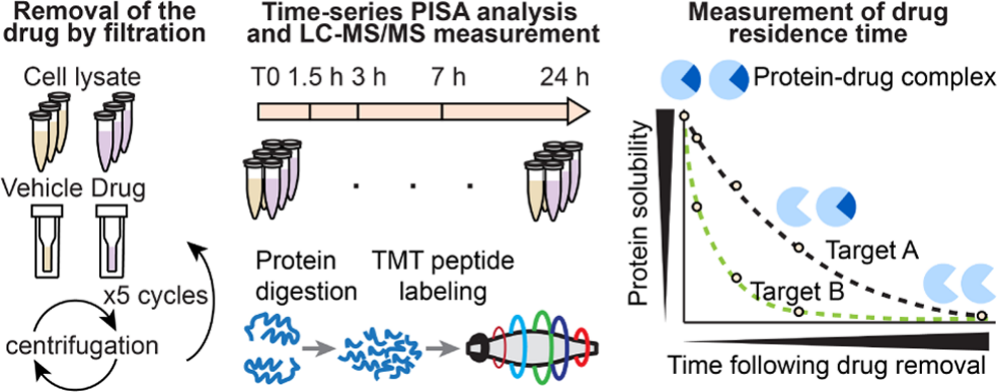

Most drugs are used in the clinic and drug candidate target multiple proteins, and thus detailed characterization of their efficacy targets is required. While current methods rely on quantitative measurements at thermodynamic equilibrium, kinetic parameters such as the residence time of a drug on its target provide a better proxy for efficacy *in vivo*. Here, we present a residence time proteome integral solubility alteration (ResT-PISA) assay, which facilitates monitoring temporal protein solubility profiles after drug removal (“off-curve”) in cell lysates or intact cells, quantifying the lifetime of drug–target interaction. A compressed version of the assay measures the integral under the off-curve enabling the multiplexing of binding affinity and residence time assessments into a single proteomic analysis. We introduce a combined scoring system for three parametric dimensions to improve prioritization of targets. By providing complementary information to other characteristics of drug–target interaction, the ResT-PISA approach will be useful in drug development and precision medicine.

The major disconnect existing between *in vitro* data on drug–target interactions and drug efficacy in humans is one of the primary sources of attrition during drug discovery.^1,2^ Traditionally, equilibrium thermodynamic constants measured *in vitro*, such as *K*_d_ and *K*_i_, have been used to assess the chances of a drug candidate for efficient target engagement *in vivo*. However, due to the biological complexity of the human organism, it is unlikely that a single *in vitro* parameter can reliably predict *in vivo* drug efficacy; a model taking into account a number of orthogonal parameters will have an *a priori* higher predictive power.

One of the critical contributors to the drug action mechanism *in vivo* is the lifetime of the drug–target complex, during which the drug molecule affects the biological system. Thus, the residence time of the drug on target, which is the reciprocal of the rate constant for dissociation of the drug–target complex, is emerging as a strong contributor to the desired predicting model of *in vivo* efficacy.^3−9^ The importance of such a kinetic parameter is due to the fact that cells, organs, and organisms are open systems operating far from equilibrium. In open systems, the concentrations of the drug, the endogenous ligands for the target, and the target itself fluctuate with time.^10^ Therefore, the residence time may correlate with *in vivo* drug efficacy better than *in vitro* equilibrium constants. Indeed, empirical observations support the notion that compounds with a longer residence time have higher *in vivo* efficacy.^11^

Another common complicating factor in drug development is that the drug usually interacts *in vivo* with several proteins, the main target as well as off-targets, with all of these molecules competing with each other for the ligand in an open system. As a drug only exerts its effect through a given target when it is bound to that target, to predict the drug efficacy in such a complex environment one has to be able to compare the lifetimes of all individual drug–target complexes. For example, the difference in the residence time of a drug on its target and on the off-target proteins responsible for toxic side effects defines to a large extent the therapeutic window of the drug.^10,12,13^

It has thus been suggested that measurements of drug–target residence time should be incorporated into the standard drug discovery procedure during the lead optimization phase.^14^ Such measurements are usually performed either with surface plasmon resonance, where a model fitted to sensogram data provides the residence time value,^15^ or with competitive binding assay using designed competitive probes.^16^ In the first case, the model assumes that drug binding to the pocket is either a simple one-step process (Figure 1a) or a more complicated two-step process. In the latter case, the initial, weaker binding at or near the pocket is followed by binding-induced structural changes in the enzyme to better accommodate the drug. This accommodation results in stronger binding and consequently longer lifetime of the drug–target complex.^17^ The corresponding “off” curves are thus either exponential or exhibit both fast and slow components (Figure 1b).

**Figure 1:**
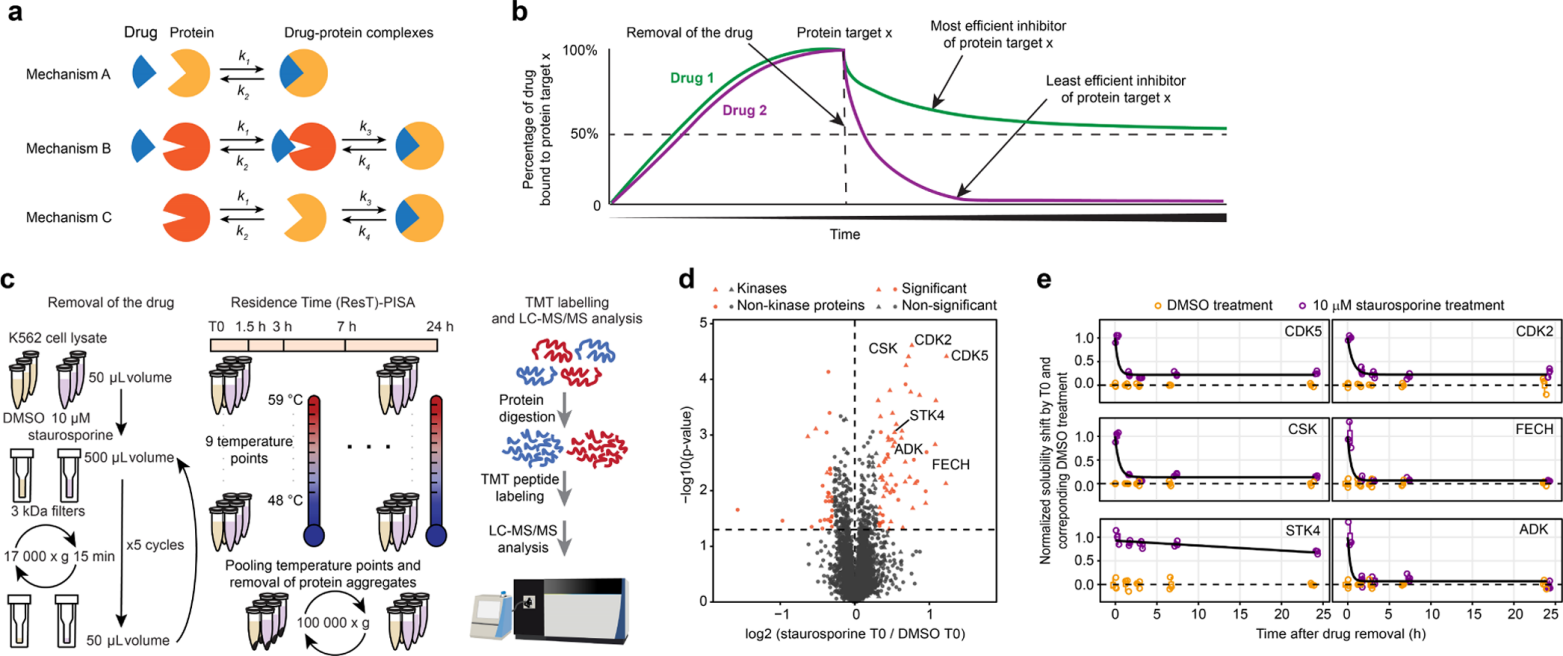
Drug–target residence time assessment using the PISA assay. (a) Different modes of ligand–protein binding. (b) Residence time of drugs can vary significantly even when dose-response are similar for two different drugs (drug 1 and drug 2). (c) Workflow of ResT-PISA analysis using staurosporine treatment of K562 cell lysate. (d) Volcano plot of protein solubility shift (ΔSm) in PISA between staurosporine and dimethyl sulfoxide (DMSO) control at the start of the experiment (T0) corresponding to the maximum drug concentration. (e) Off-curves for selected targets show significant solubility changes with time past staurosporine removal. Horizontal lines in the boxplots represent the median, 25th and 75th percentiles and whiskers represent measurements to the 5th and 95th percentiles. (*n* = 3). P-values were calculated using a two-tailed Student’s *t*-test.

The above approaches are developed for studying simple systems with highly purified recombinant targets and drug molecules often modified for targeted purification or better detection. There exist proteome-wide methods of probing protein–target interactions not requiring drug modification, such as the cellular thermal shift assay (CETSA),^18^ thermal proteome profiling (TPP),^19^ as well as drug affinity responsive target stability (DARTS)^20^ and LiP-MS.^21,22^ These methods have been extensively used to deconvolute drug targets in cell lysates, intact cells, body fluids, and even tissues. However, all of these methods suffer from high analysis costs and low throughput. Some time ago, we have introduced the proteome integral solubility alteration (PISA) assay as a high-throughput analogue of TPP.^23^ PISA analysis was used for deconvoluting the action mechanism of redox modulating anti-cancer compounds^24^ and profiling stem cell transitions.^25^ Here, we hypothesize that the ΔSm solubility shift in PISA will reflect the degree of target engagement by a drug in a cell lysate or intact cell at a certain time past drug removal by filtration (for lysate) or washing (for cells). If so, the high-throughput nature of PISA analysis should allow us to probe the residence time of a drug through the temporary profiles of ΔSm for different targets and off-targets within the same proteome. We also hypothesize that the obtained residence times will be independent of the maximum ΔSm values that reflect the effect of drug binding on target solubility (sometimes interpreted as thermal stability^19^). The mutual independence of these parameters would mean that the addition of the residence time information to ΔSm data would augment the model predicting *in vivo* drug efficacy.

Here, we test the above hypotheses by developing a novel technique called residence time proteome integral solubility alteration (ResT-PISA) assay. We show that most, but not all, protein targets of staurosporine recover their solubility after a short period of time following drug removal, providing a proof of principle for ResT-PISA. Thereafter, we verify that even covalent binding of a simple chemical group results in a complex residence time behavior reflecting both short-term binding as well as long-lasting interaction. To increase the throughput of our method, we compress ResT-PISA to cResT-PISA analysis and include dose-response measurements (conc-PISA) in one multiplexed sample. This allowed us to create a scoring system based on three parameters ΔSm, ResT, and dose-response, to improve characterization and prioritization of efficacy targets. Finally, we show that ResT-PISA is amenable to intact cells. Our new approach for evaluating drug–target residence time in cell lysates and intact cells using an unbiased, label-free, and system-wide method can find applications in drug discovery and development, as well as in personalized medicine.

## Materials and Methods

Detailed experimental information including cell culture, PISA assay, protein sample preparation for expression proteomics and TMT labeling, high pH reversed-phase peptide fractionation, and bioinformatics analysis^38,39^ are provided in the Supporting Information PDF file.

### Sample Preparation for ResT-PISA in the Cell Lysate

K562 cells were grown until they reached a concentration of around 1 × 10^6^ cells/mL. Then 40 mL of cell culture was pelleted at 400*g* for 3 min and washed with phosphate-buffered saline (PBS); the operation was repeated twice. Cells were pelleted one last time at 400*g* for 5 min and resuspended in 5 mL of PBS (GIBCO) supplemented with protease inhibitors (ThermoFisher Scientific). The cell lysate was clarified by centrifugation at 20 000*g* for 5 min and the supernatant was collected. Then, 100 μL of the supernatant were aliquoted in 30 PCR tubes, half were treated at a concentration of 10 μM staurosporine (Selleckchem), 2 μM ponatinib (Selleckchem) or 2 μM iodoacetamide (IAA) (Sigma-Aldrich), the other half was treated with a vehicle (DMSO or water) and maintained at 37 °C for 45 min. Then, 50 μL of each aliquot was transferred to a 500 μL 3 kDa filtration unit (Merck) and immediately 450 μL of PBS supplemented with protease inhibitors were added to each aliquot, with 3 aliquots receiving additionally the same initial concentration of a drug and 3 aliquots receiving the same concentration of vehicle instead. Then the filtering units were centrifuged at 17 000*g* for 15 min and 450 μL of the same solutions were added on top of each sample (10 × dilution). The operation was repeated 6 times. PISA assay was then performed on each sample at different time points, with T0 corresponding to the samples that were filtered with the addition of a drug. Given the time required for centrifugation and aliquoting for PISA assay (see below), the first time point was 1.5 h after the start of drug removal. These samples were processed in parallel with the samples that received drug and DMSO during filtration (T0) for PISA analysis. The rest of the samples were left at RT for 3, 7, and 24 h from the start of drug removal, and then were subjected to PISA analysis.

### Sample Preparation for ResT-PISA in Cells

K562 cells were grown in IMDM for SILAC (ThermoFisher Scientific) supplemented with either heavy (Cambridge Isotope Laboratories) or light (Sigma-Aldrich) lysine and arginine and 10% dialyzed FBS (ThermoFisher Scientific). Cells were grown until they reached a concentration of around 1.10^6^ cells/mL. The cells were split into two groups in 75 cm^2^ culture flasks (Sarstedt), the first group corresponding to heavy SILAC was treated for 1.5 h using 2 μM staurosporine and the other group corresponding to light SILAC was treated with an equivalent concentration of vehicle (DMSO) (Figure 5a). For T0, triplicates of staurosporine or DMSO treatment were deposited in individual tubes and rinsed three times with PBS containing protease inhibitors as well as the corresponding concentration of staurosporine or DMSO, to control for potential changes occurring in the samples during the centrifugation steps. For the samples corresponding to the 20 min time point, the cells were deposited in tubes in triplicates and rinsed three times with PBS containing protease inhibitors. Both cells from T0 and 20 min time point were then distributed into PCR tubes for PISA assay (see below). For longer time points, cells treated with staurosporine or DMSO were rinsed three times with the fresh medium. Then the cells were seeded into 25 cm^2^ culture flasks (Sarstedt) in triplicate for each treatment and time point and placed again in the cell incubator for the intended duration. Then the cells previously treated with staurosporine or DMSO were rinsed three times with PBS, aliquoted into PCR tubes, and processed for PISA assay (see the Supporting Information).

### Compressed ResT-PISA

For cResT-PISA, ResT-PISA analysis in K562 cell lysate was performed as described above with the only difference being that after heating the aliquots and snap-freezing, the temperature and time points corresponding to one replicate of ResT-PISA were all pooled together, which resulted in 9 samples: 3 × 2 μM ponatinib treatment, 3 × DMSO treatment, and 3 × of the pooled ResT-PISA range (cResT-PISA).

### Dose-Response PISA (conc-PISA)

In parallel, we performed conc-PISA analysis as previously described.^23^ Briefly, the same K562 cell lysate was aliquoted into 12 samples per replicate and treated with the following concentrations for 45 min: 2 samples with 2 μM, 2 samples with DMSO only, one sample each with 2 pM, 20 pM, 200 pM, 2 nM, 20 nM, and 200 nM. Then, each sample was aliquoted into 9 tubes in triplicate and each replicate was heated for 3 min to the same 9 temperature points as in ResT-PISA and then left at RT for 3 min and snap-frozen.

After this, all 9 samples in each replicate were pooled together into a conc-PISA replicate. For the maximal and minimal drug concentration, only the samples corresponding to the treatment with 2 μM ponatinib and DMSO were pooled together. This resulted in 9 samples in total: 3 × 2 μM ponatinib treatment, 3 × DMSO treatment, and 3 × of the pooled concentration range (conc-PISA). After pooling the samples were processed as in PISA analysis (centrifuged and digested for liquid chromatography and tandem mass spectrometry (LC-MS/MS) analysis).

### Mass Spectrometry Analysis

Each sample was resuspended in 2% ACN and 0.1% FA at a concentration of 0.2 μg/μL and 1 μg was injected into the respective LC system (summarized in Table S5). Mass spectra were acquired using the parameters listed in Table S5.

### Mass Spectrometry Data Analysis

Raw files were converted to the mzML format using MSConvert (version 3.0.21258).^34^ Peak picking of profile spectra was performed with the vendor-provided algorithm (ThermoFisher Scientific). Then, individual datasets were searched using FragPipe GUI v17.1 with MSFragger (version 3.4)^35^ as the search algorithm. Protein identification was performed with the human Swisspot database (20′409 entries, downloaded on 2022.02.22), with acetylation (N-terminus) and oxidation on methionine as variable modification and carbamidomethylation of cysteine residues, TMT or TMTpro on the N-terminus or lysine as fixed isobaric labels. Trypsin was set as the enzyme with up to two missed cleavages. The peptide length was set to 7–50, and peptide mass range of 200–5000 Da. For MS2-based experiments, the precursor tolerance was set to 20 ppm and fragment tolerance to 20 ppm. Peptide spectrum matches (PSMs) were adjusted to a 1% false discovery rate using Percolator^36^ as part of the Philosopher toolkit (v4.1.0+).^37^ For TMT labeled samples, reporter intensities were extracted by TMT-Integrator with default settings. As TMTpro18-plex labeling was not supported, reporter intensities were extracted by a home-written algorithm in R (version 4.1.1) as follows: 20 ppm windows around the theoretical *m*/*z* of each reporter ion were investigated and the abundance of the most intense peak was extracted. For SILAC-TMT, all settings were the same, except for the fragment tolerance that was set to 0.6 Th; also, heavy lysine (+8.014199 Da) and arginine (+10.008269 Da) were added as variable modifications.

### ResT-PISA Data Analysis

Protein abundance at each time point was normalized by the corresponding protein abundance in the vehicle-treated sample at the same time point and statistical analysis was performed with a two-tailed *t*-test with equal variance. For visualization purposes, only proteins with a log2-scaled abundance fold change of at least 0.3 and a *p*-value below 0.05 were considered to be altered and used for further analysis of the residence time.

For curve fitting of residence time data, protein fold changes were first scaled to the mean fold change of the drug-treated sample at time 0. Then, if the mean of the drug-treated sample at the last time point was below 0.6, an exponential off-curve with an asymptotic term:
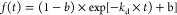
was fitted. If not, then a linear function
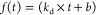
was fitted, where *b* is the asymptotic term and *k*_d_ is the rate constant for protein–drug binding.

Area under the curve (AUC) calculations were made by summing for each replicate the mean of all data points over the entire time course.

Full curve ResT-PISA data in intact cells were treated in the same way with the exception that changes in the protein expression were accounted for by normalizing each time point by their corresponding expression value.

All correlations were calculated as Pearson’s linear correlations.

### Compressed Concentration and Residence Time of PISA

In both experiments, the protein abundance changes were normalized by those of the corresponding vehicle-treated samples. The significance of abundance changes was calculated as above. For further analysis the significant proteins from the maximal drug concentration or T0, for conc-PISA and cResT-PISA, respectively, were selected for analysis and protein solubility alterations were first scaled to the mean of their respective PISA samples treated with either ponatinib or DMSO. Then, proteins with a standard deviation between the replicates at the maximal concentration or the first-time point bigger than three times the maximal mean value were removed. Finally, proteins that had a scaled conc-PISA or ResT-PISA value above 1 or below 0 were removed.

The combined score for the filtered proteins was calculated as follows: (1) the scaled protein solubility alteration was determined by scaling each protein mean solubility alteration at the maximal drug concentration by the biggest absolute value among these proteins (alteration score or Scaled ΔSm), (2) the scaled drug concentration value was calculated as described in (1) for conc-PISA values (concentration.score or conc-PISA), and (3) the scaled residence time value was calculated as described in (1) for cResT-PISA values (residence time.score or cResT-PISA). The final score was calculated as the sum of the scores in (1), (2), and (3).

## Results and Discussion

### ResT-PISA Assay Estimates Drug–Target Residence Times in a Complex Protein Mixture

As a way of obtaining a proof of principle for ResT-PISA, we asked the question of whether the multiple targets of a promiscuous drug will show different residence times in ResT-PISA analysis. To this end we treated a K562 cell lysate with 10 μM staurosporine chosen as the model drug known to inhibit many protein kinases.^19^ We removed the drug after 45 min of treatment using 5 successive filtrations through 3 kDa membrane filters (approximately 1.10^6^ × dilution) (Figure 1c). We opted for 3 kDa filters to minimize protein losses (Figure S1a). Then we performed PISA analysis right after filtration (which corresponds to 1.5 h time interval from the start of the filtration process), as well as after 3, 7, and 24 h. To obtain a measure of the effect of the maximal drug concentration on the solubility of protein targets, we analyzed by PISA a lysate treated with staurosporine and filtered with the addition of PBS containing the same initial concentration of staurosporine (10 μM), in comparison with a control treated with vehicle (DMSO) instead of the drug (Figure 1c). PISA measurements were also used to normalize the ResT-PISA results after removal of the drug. We recorded the off-curves for 5556 proteins quantified across all samples without missing values, among which 47 kinases showed significant solubility alteration after treatment with staurosporine at T0 (Figure 1d, Table S1). Most of these proteins recovered their solubility to the levels of an untreated sample after a short period of time past drug removal (Figure 1e). These recovered proteins include multiple kinases, such as CDK2 and CDK5, and a common non-kinase off-target of kinase inhibitors, Ferrochelatase (FECH).^19,26^ However, some proteins like STK4 recovered less than half of their solubility changes in 24 h after drug removal, suggesting a strong or covalent interaction (Figure 1e).

### ResT-PISA Analysis Can Detect Binding Events with Prolonged Lifetimes

Being intrigued by the remarkably slow recovery of the staurosporine target STK4, we decided to investigate the off-curves of covalently attaching molecules. To this end, we performed a ResT-PISA experiment in the K562 cell lysate with 2 μM iodoacetamide (IAA) instead of staurosporine. IAA is widely used in proteomics to stabilize reduced cysteines by covalently adding to them a carbamidomethyl group. Since such alkylation reaction targets all accessible free cysteines, we expected to observe significant solubility shifts ΔSm for many proteins. We also expected that most of these proteins will show no or minimal recovery of solubility hours after the removal of the chemical due to the covalent nature of the modification.

**Figure 2:**
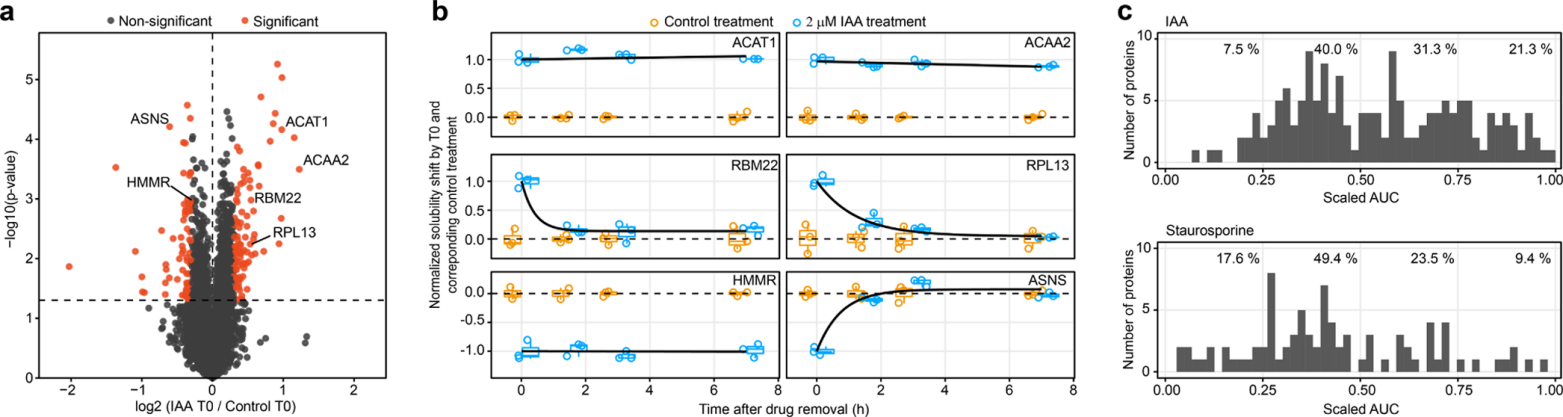
ResT-PISA of IAA as a covalent modifier. (a) Volcano plot of protein solubility shift between IAA and water control at the start of the experiment (T0) corresponding to the maximum compound concentration. (b) Off-curves for selected targets show a significant solubility change with time past IAA removal. Horizontal lines in the boxplots represent the median, 25th and 75th percentiles and whiskers represent measurements to the 5th and 95th percentiles. (c) Histograms of areas under the curves (AUCs) in (b) normalized by the maximum AUC value (*n* = 3). *P*-values were calculated using a two-sided Student’s *t*-test.

The results showed that the solubility was significantly affected by IAA in 165 proteins, of which 67 proteins lowered their solubility and 98 proteins (59%) increased it. While some of these proteins demonstrated, as expected, no solubility recovery 7 h after drug removal (Figure 2a,b, Table S2), some of the proteins did recover their initial solubility, suggesting that they either lost their cysteine modification or IAA was binding to them noncovalently (such binding is often the first step before covalent attachment). This observation highlights the complexity of protein-small molecule interactions that can occur through different modalities, as depicted in Figure 1a. Interestingly, some of the targets of IAA with high ΔSm values and long residence times include two acetyl-COA acetyltransferases ACAT1 and ACAA2 (Figure 2a,b). Since IAA has been used to inhibit glycolysis,^27^ and as ACAT1 activity is linked to the Warburg effect in cancer cells,^28^ inhibition of acetyl-COA acetyltransferases may at least in part be responsible for IAA-induced reduction in glycolytic activity.

**Figure 3:**
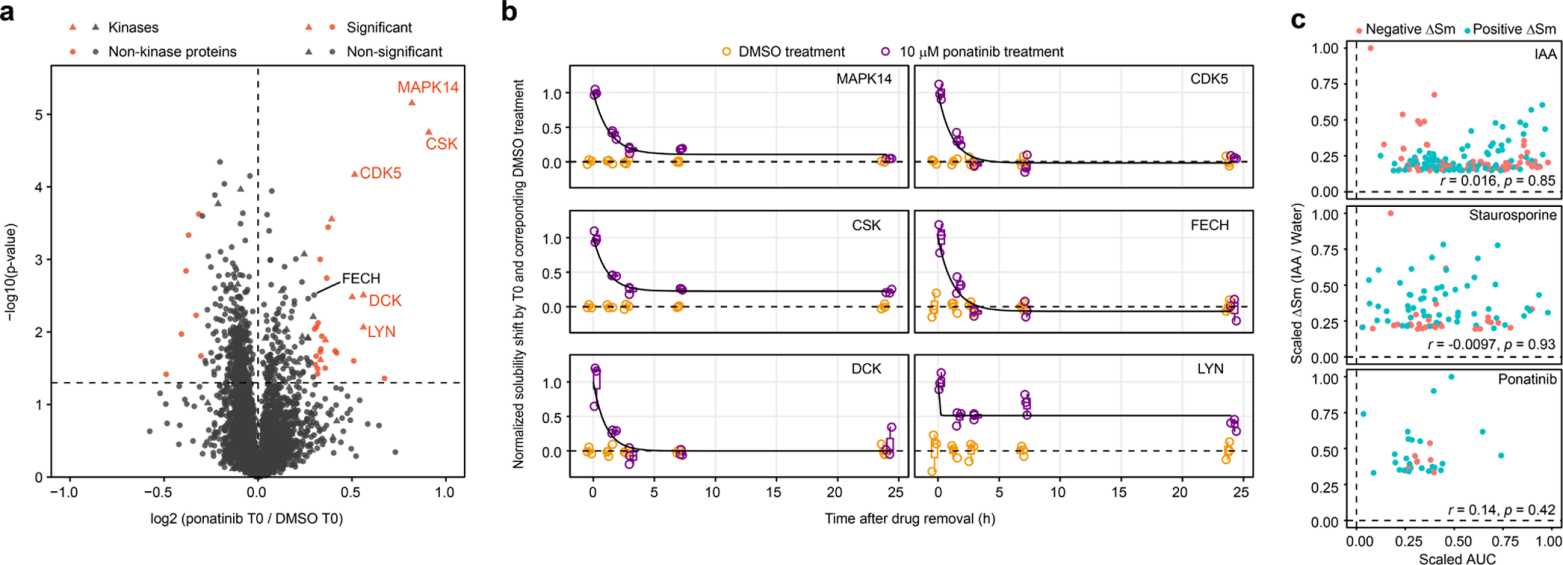
ResT-PISA of a kinase inhibitor ponatinib and comparison of AUCs with ΔSm. (**a**) Volcano plot of protein solubility shift between ponatinib and DMSO control at the start of the experiment (T0) corresponding to the maximum drug concentration. (**b**) Off-curves for selected targets show a significant solubility change with time past ponatinib removal. Horizontal lines in the boxplots represent the median, 25th and 75th percentiles and whiskers represent measurements of the 5th and 95th percentiles. (**c**) Residence time (*y*-axis) versus the scaled solubility shift ΔSm (*y*-axis) for proteins with shifting solubility in IAA, staurosporine, and ponatinib treatments. Pearson correlations between the two measures were calculated for each treatment, *n* = 3. *P*-values were calculated using a two-sided Student’s *t*-test.

To get further insight into the ResT-PISA specifics of the covalently attaching IAA and noncovalently interacting staurosporine, we compared for both drugs the distributions of the normalized areas under the curve (AUCs) that scale for a given drug with its residence time on a target protein. While AUCs of the majority (53%) of the detected targets of IAA scaled above 0.5, indicating slow-unbinding targets, for staurosporine the corresponding figure was significantly lower, 30% (Figure 2c). Next, we tested ponatinib, a multi-target kinase inhibitor used in clinics for the treatment of chronic myeloid leukemia and Philadelphia chromosome-positive acute lymphoblastic leukemia.^29^ PISA results revealed solubility shifts in multiple kinases previously reported as targets of ponatinib, such as LYN, MAPK14, CSK, IRAK4, and RIPK2,^30^ as well as in kinases that have not yet been reported as ponatinib targets (Figure 3a, Table S3). Additionally, we detected a significant solubility shift for FECH. The residence time was assessed for all significantly shifting proteins as AUC of their off-curves (Figure 3b). For all three molecules tested until this point, ΔSm did not correlate with the residence times of the proteins with shifting solubility (Figure 3c): the Pearson correlations of 0.02, −0.01, and 0.14 proved that these two parameters are largely independent. This result supported our hypothesis that residence time can be used as an independent kinetic parameter for target prioritization. Although protease inhibitors were present in the lysis buffer to prevent protein degradation, it could still occur during long incubation at room temperature. To verify that protein degradation over time is not responsible for the observed recovery in protein solubility, we plotted the distribution of the protein abundances in the controls at each time point and did not observe an increased standard deviation between the replicates over time in the DMSO control (Figure S1b). In addition, since we normalized protein abundances in each treated sample by the time-matched control, degradation effects would cancel out. Thus, the recovery of protein solubility over time is likely to originate from the drug detaching from the protein target rather than being an artifact of sample treatment.

Finally, we investigated whether the measurements from ResT-PISA are in accordance with the published datasets containing binding kinetics data for staurosporine and ponatinib^15,16^ obtained using surface plasmon resonance and a kinetic probe competition assay. In total, 6 kinases present in these studies are significantly shifting and have a fitted curve in our dataset. Most of our measurements are in a qualitative agreement with the published residence times (Figure S1c). When there is a discrepancy between the two studies regarding ponatinib’s residence time on LYN, our estimate of ≈22 h is closer to 12.9 h of Willemsen-Seegers et al. than to 33 min of Georgi et al. It should be noted that both cited studies used a short time scale of measurements (20 min maximum), which resulted in an increased error for time constants derived from slowly decaying off-curves. On the contrary, ResT-PISA estimates best the curves lasting 10 min and longer and is presently incapable of measuring on the time scale of seconds. Moreover, the cited studies used purified proteins, while ResT-PISA deals with complex lysates and living cells. Thus, ResT-PISA is a complementary approach to many existing methods.

### Off-Curve Measurements in ResT-PISA Can be Compressed

**Figure 4:**
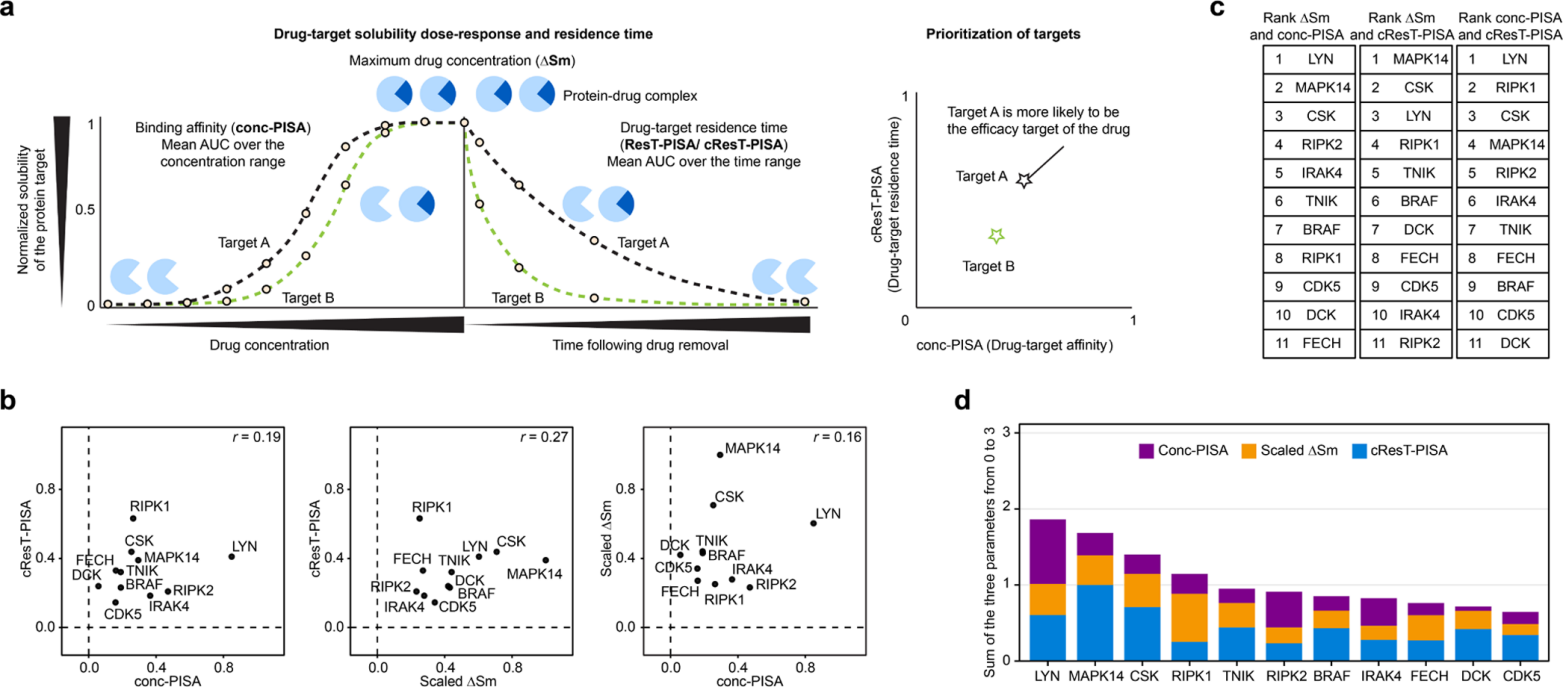
Combining PISA, cResT-PISA, and conc-PISA results in a single score for prioritization of drug targets. (a) Example of comparing the dose responses and residence times and subsequent prioritization of two protein targets. (b) Plots of ΔSm against cResT-PISA, ΔSm against conc-PISA and cResT-PISA against conc-PISA for selected targets of ponatinib in K562 cell lysate. Pearson’s correlations were calculated for each plot. (c) Ranking of selected targets of ponatinib for ΔSm, cResT-PISA and conc-PISA scaled to the interval between 0 and 1. (d) Ranking of each target of ponatinib according to their combined scores.

We investigated whether the ResT-PISA analysis could be further compressed by pooling all time points together during sample preparation. This approach is similar to the one used in PISA in that it provides an AUC directly from one measurement. We called this approach compressed ResT-PISA or cResT-PISA. Since cResT-PISA can produce triplicate analysis using only 9 samples (3 × PISA at T0, 3 × Control, 3 × cResT-PISA), we could multiplex two cResT-PISA analyses in a single TMT18-plexed set. The AUC from cResT-PISA correlated well (*r* = 0.79) with the ResT-PISA AUCs for protein kinases (Figure S2a), confirming that pooling the samples preserves residence time information. Alternatively, we could multiplex with cResT-PISA altogether different measurements, such as the dependence of ΔSm upon drug concentration (Figure 4a, Table S3). Earlier we showed that this dose–response curve can also be compressed in a PISA-like fashion;^23^ here, we compressed in “conc-PISA” 10 concentration points starting from 2 μM and proceeding with a 10-fold dilution at every successive concentration point. The results of conc-PISA measurements provide a proxy for the association rate constant *k*_on_. It is important to note that the accuracy of the residence time and concentration dependence estimates is higher for proteins that show large ΔSm shifts (Figure S2b).

Then, we could multiplex in a TMT18 set three replicates of conc-PISA and cResT-PISA analysis in K562 cell lysate. Each TMT-multiplexed conc-PISA sample contained: 3 × control PISA samples (incubation of lysate with DMSO for 45 min), 3 × PISA samples incubated with ponatinib for 45 min at a maximum concentration of 2 μM (as in a regular PISA analysis), and 3 × PISA of the samples incubated with ponatinib for 45 min in a range of 10 concentrations (See Materials and Methods) pooled together, while each cResT-PISA sample contained: 3 × control PISA samples (incubation of lysate with DMSO for 45 min) filtered through 3 kDa filters with a buffer containing DMSO (see Materials and Methods), 3 × PISA of samples incubated with ponatinib for 45 min at a maximum concentration of 2 μM and then filtered through 3 kDa filters in a buffer containing 2 μM of ponatinib, and finally 3 × PISA of the samples incubated with 2 μM ponatinib for 45 min, followed by drug removal by spinning through a 3 kDa filter in a buffer without drug or DMSO with a varying time delay upon removal of the drug (0, 1.5, 3, 7, and 24 h).

### Combining PISA, cResT-PISA, and conc-PISA Results in a Single Score for Target Prioritizing

We compared the relative solubility shifts in PISA, cResT-PISA, and conc-PISA and found no apparent correlation between them, confirming that these parameters are largely independent (Figure 4b). Rankings of each target by the corresponding parameter are shown in Figure 4c. To combine the relative contribution of each parameter, we scaled each of them to a minimum of 0 and a maximum of 1 and summed the results to obtain a final score and overall target ranking. The known target of ponatinib ABL1 was not among the top-ranked proteins, as we did not detect significant solubility shift in that protein. This could be due to the fact that K562 cells express a BCR-ABL fusion protein which earlier showed no ΔTm shift in TPP.^19^ Instead, LYN kinase, a well-known target of ponatinib, ranked first. LYN is engaged by ponatinib at one of the lowest concentrations among known ponatinib targets;^30^ in addition, it is known to produce a substantial thermal stability shift in TPP.^30^ LYN was found by ResT-PISA to associate with ponatinib for a prolonged time, which explains why this protein came up as the top target in our analysis. Two other top proteins in overall ranking are also known targets MAPK14 and CSK that engage ponatinib at low concentrations and exhibit large thermal stability shifts in TPP.^30^

### Visualization Tool for X-PISA Analyses

To facilitate analysis of a combined set of PISA data (PISA, cResT-PISA, and conc-PISA) we designed a data processing and visualization tool that is available on Github (https://github.com/RZlab/PISA-Analyzer). The user guide to the interface is provided in Figure S3.

### ResT-PISA is Amenable to Intact Cells

**Figure 5:**
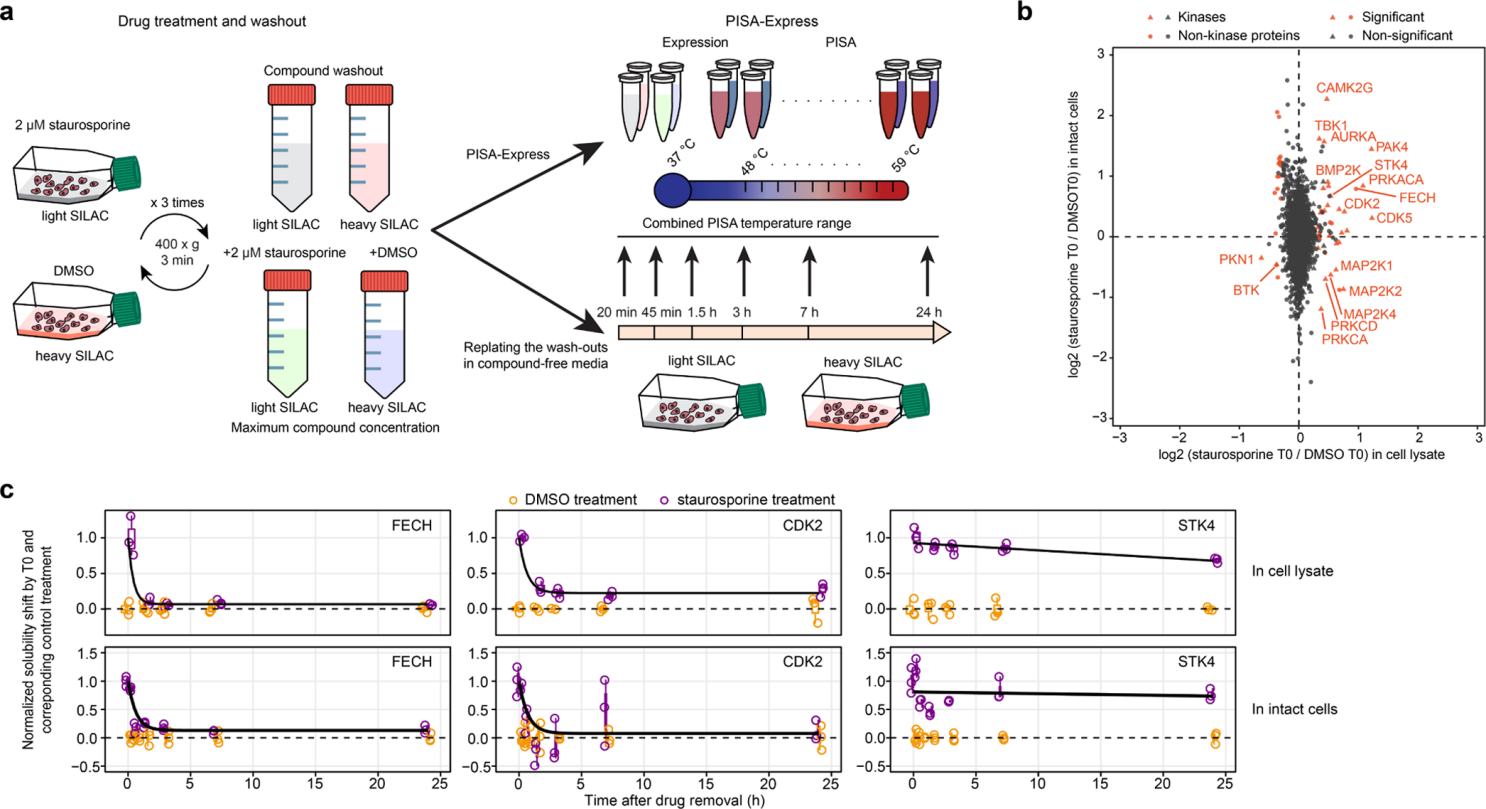
ResT-PISA in intact cells. (a) In-cell ResT-PISA workflow. (b) Comparison of ΔSm at T0 in K562 cell lysate (*x*-axis) and intact cells (*y*-axis) for staurosporine treatment against DMSO control. (c) Off-curves for three selected targets, showing a significant solubility shift upon staurosporine removal in both cell lysate and intact cells. Horizontal lines in the boxplots represent the median, 25th and 75th percentiles and whiskers represent measurements to the 5th and 95th percentiles. *n* = 3. *P*-values were calculated using a two-sided Student’s *t*-test.

Unlike cell lysate, a living cell actively imports, metabolizes, and exports drugs and their metabolites, and thus the ability to monitor drug–target residence time in a cellular environment would be very valuable for predicting *in vivo* drug efficacy. Thus, we performed ResT-PISA in intact K562 cells treated with staurosporine and compared the outcome with the lysate results. Since the removal of the drug from the growth media is a simple washing procedure, we could start recording residence time as early as 20 min past drug removal, i.e., much faster than that with a lysate (Figure 5a). In addition, the cell experiment did not require extensive filtering that may result in protein loss. On the other hand, the cell experiment required normalization of each PISA dataset by separately measuring protein abundance, as in PISA-Express,^25^ and thus demands multiplexing of a larger number of samples. To meet this increased demand, we metabolically labeled proteins in cells by light and heavy stable isotope labeling by amino acids in cell culture (SILAC)^31^ reagents and additionally used TMT16-plex labeling of tryptic peptides. We performed PISA-express analysis at each time point using an expression control at T0 as well as after 1.5, 3, 7, and 24 h past drug removal. The obtained thermal shifts as well as residence times provided a detailed picture of drug effects in intact cells (Figure 5a).

Surprisingly, some of the solubility shifts observed in lysate occurred in opposite directions (Figure 5b, Table S4); this was the case for MAP2K1, MAP2K2, MAP2K4, PRKCA, and PRKCD. MAP2Ks both phosphorylate kinases are phosphorylated by kinases in signaling cascades involved in stress response.^32^ PRKCA is a calcium-activated and diacylglycerol-dependent protein, while PRKCD is phospholipid and diacylglycerol-dependent; both proteins are involved in several regulatory processes including regulation of apoptosis.^33^ These features may explain the difference in the direction of the solubility alteration in cells and lysates. When considering the absolute values of the solubility shifts, a positive correlation between the ResT-PISA AUCs in lysate and intact cells for common kinases was observed (*r* = 0.59), while for all common proteins there was an anti-correlation (*r* = −0.39) (Figure S4).

Many proteins showed similar residence times in cell lysate and intact cells (Figure 5c). While for fast unbinding in lysate targets, such as FECH and CDK2, this result could be expected, it came as a surprise for slow-unbinding targets, as the intact cell could detoxify the drug metabolically and/or degrade and replace the inhibited protein within hours past drug removal. However, STK4, which showed little recovery of solubility in lysate 24 h after removal of the drug had a similar behavior in intact cells, suggesting that STK4 stayed all this time bound with staurosporine and avoided being replaced in the cellular environment.

## Conclusions

Here, we developed ResT-PISA and its compressed version cResT-PISA, a compact approach to measuring drug–target residence times at the proteome level in both cell lysate and intact cells. As well as other PISA-based techniques, ResT-PISA does not require drug modification or the use of recombinant proteins. Importantly, the ResT-PISA approach is not limited to a selected class of compounds, as e.g., the competitive probes method that targets proteins with defined activity, such as kinases. Being multiplexed with PISA and concentration-dependent PISA analyses, this approach provides a comprehensive assessment of target engagement, binding affinity, and residence time simultaneously. This enables extensive characterization of drug properties in a single analysis and provides a combined score for target prioritization. As a limitation, very short time frames (seconds or milliseconds) are not currently available due to the longer time required to remove the drug through cell washing or lysate filtration. Another limitation ResT-PISA suffers from is the same as in CETSA, TPP, and PISA, since some targets exhibit upon drug binding the solubility changes that are too subtle to be detected with statistical significance, as extensively discussed in the literature.^18,19,24,40−43^ This drawback can at least partially be alleviated by increasing the statistical power of analysis by employing larger number of replicates. Finally, protein solubility shifts may not always originate from direct binding, e.g., when the drug stabilizes the whole protein complex while binding to only one of its components.

## Supporting Information Available

Protein mass distribution, distribution of protein abundances in control samples over time, and comparison of residence time measurements from other studies (Figure S1); correlation between the theoretical compressed ResT-PISA of ponatinib targets versus the observed experimental values and relation between the ΔSm and standard deviation in cResT-PISA and conc-PISA (Figure S2); overview of the PISA-analyzer interface (Figure S3); correlation of ResT-PISA of staurosporine in lysate and in cell (Figure S4). LC-MS/MS systems and parameters. Detailed experimental procedure (Table S5) (PDF)
ResT-PISA of staurosporine in K562 cell lysate (Table S1) (XLSX)
ResT-PISA of IAA in K562 cell lysate (Table S2) (XLSX)
ResT-PISA, cResT-PISA, and conc-PISA of ponatinib in K562 cell lysate (Table S3) (XLSX)
ResT-PISA of staurosporine in intact K562 cells (Table S4) (XLSX)

## Notes

The mass spectrometry proteomics data have been deposited to ProteomeXchange Consortium (http://proteomecentral.proteomexchange.org) via the PRIDE partner repository with dataset identifier PXD034351. The code for the web-interface is available on GitHub/RZlab (https://github.com/RZlab/PISA-Analyzer).
