# Supplementary material for: System-Wide Profiling by Proteome Integral Solubility Alteration Assay of Drug Residence Times for Target Characterization
Source: Anal Chem. 2022 Nov 3;94(45):15772–80. doi: 10.1021/acs.analchem.2c03506 (PMC9670035; doi:10.1021/acs.analchem.2c03506)
Supplement: Supplementary file 1 — ac2c03506_si_001.pdf [file ac2c03506_si_001.pdf]

Supporting Information

System-wide profiling by proteome integral solubility alteration assay of drug residence times for target characterization

Pierre Sabatier[†,‡,§,#], Christian M. Beusch[†,#], Zhaowei Meng[†], Roman A. Zubarev[†,‖,⊥*]

[†]Division of Chemistry I, Department of Medical Biochemistry and Biophysics, Karolinska Institutet, Stockholm, 17177, Sweden. [‡]Department of Surgical Sciences, Uppsala University, Uppsala, 751 85, Sweden. [§]Novo Nordisk Foundation Center for Protein Research, University of Copenhagen, Copenhagen, 2200, Denmark. [‖]Department of Pharmacological & Technological Chemistry, I.M. Sechenov First Moscow State Medical University, Moscow, 119146, Russia. [⊥]The National Medical Research Center for Endocrinology, Moscow, 115478, Russia.

Table of Content

Figure S1. Protein mass distribution, distribution of protein abundances in control samples over time and comparison of residence time measurements from other studies.

Figure S2. Correlation between the theoretical compressed ResT-PISA of ponatinib targets versus the observed experimental values and relation between the ΔSm and standard deviation in cResT-PISA and conc-PISA.

Figure S3. Overview of the PISA-analyser interface.

Figure S4. Correlation of ResT-PISA of staurosporine in lysate and in cell.

Extended material and methods

Table S5. LC-MS/MS systems and parameters.

Supplementary figures

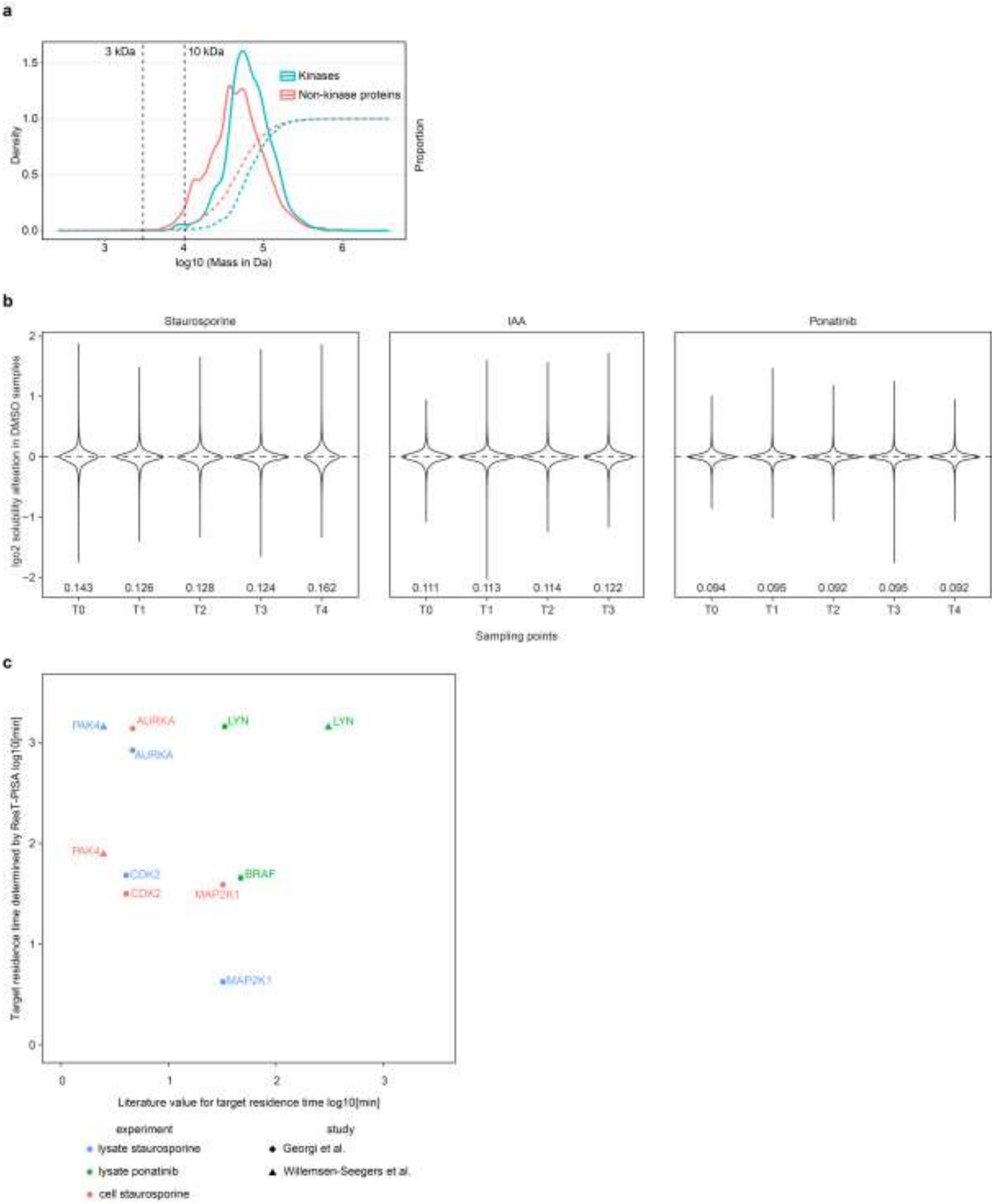

Figure S1. Protein mass distribution, distribution of protein abundances in control samples over time and comparison of residence time measurements from other studies. (**a**) Distribution of the molecular mass of protein kinases and other proteins (solid lines) and their proportion (dashed lines). The 3 kDa cutoff used for filtration for drug removal in this study is highlighted as well as another popular 10 kDa cutoff. (**b**) Distribution of the mean log2-

scaled FC of the protein abundance in each of the triplicate measurement of the control sample divided by the mean abundance of these same triplicate measurements, for each experiment at each time point. The standard deviation of each distribution is also calculated. (**c**) Comparison of the residence time estimate from Georgi et al.[15] and Willemsen-Seegers et al.[16] (x axis) and the residence time estimate from ResT-PISA (y axis) of several kinases.

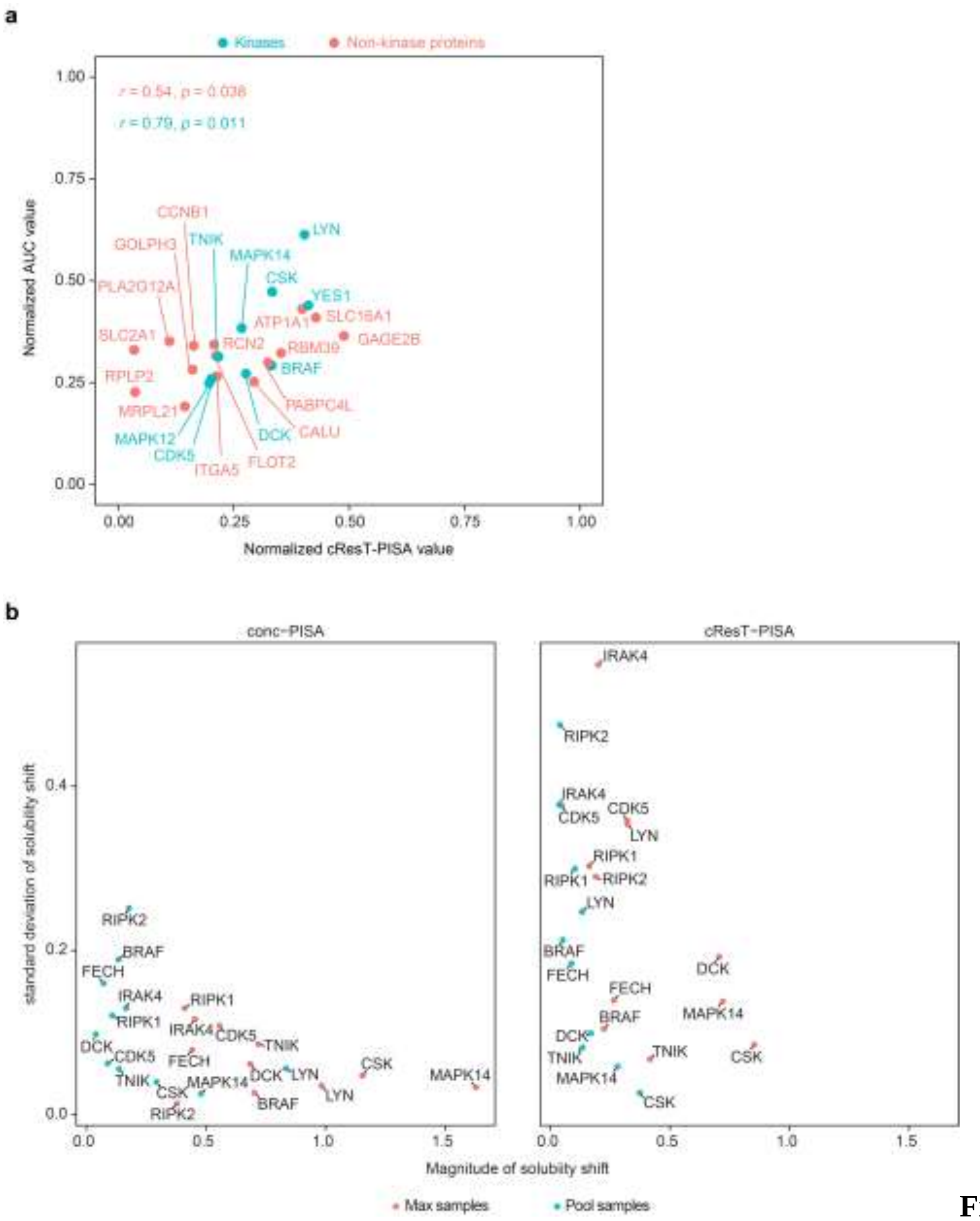

Figure S2. Correlation between the theoretical compressed ResT-PISA of ponatinib targets versus the observed experimental values and relation between the ΔSm and standard deviation in cResT-PISA and conc-PISA. (**a**) Protein kinases are shown in cyan and non-kinase proteins are shown in red. Pearson's correlations between the two measures were calculated for the two protein groups. (**b**) Relation between the magnitude of the ΔSm (x-axis) and the standard deviation in the estimation of the residence time (cResT-PISA) or concentration dependence (conc-PISA) (y-axis).

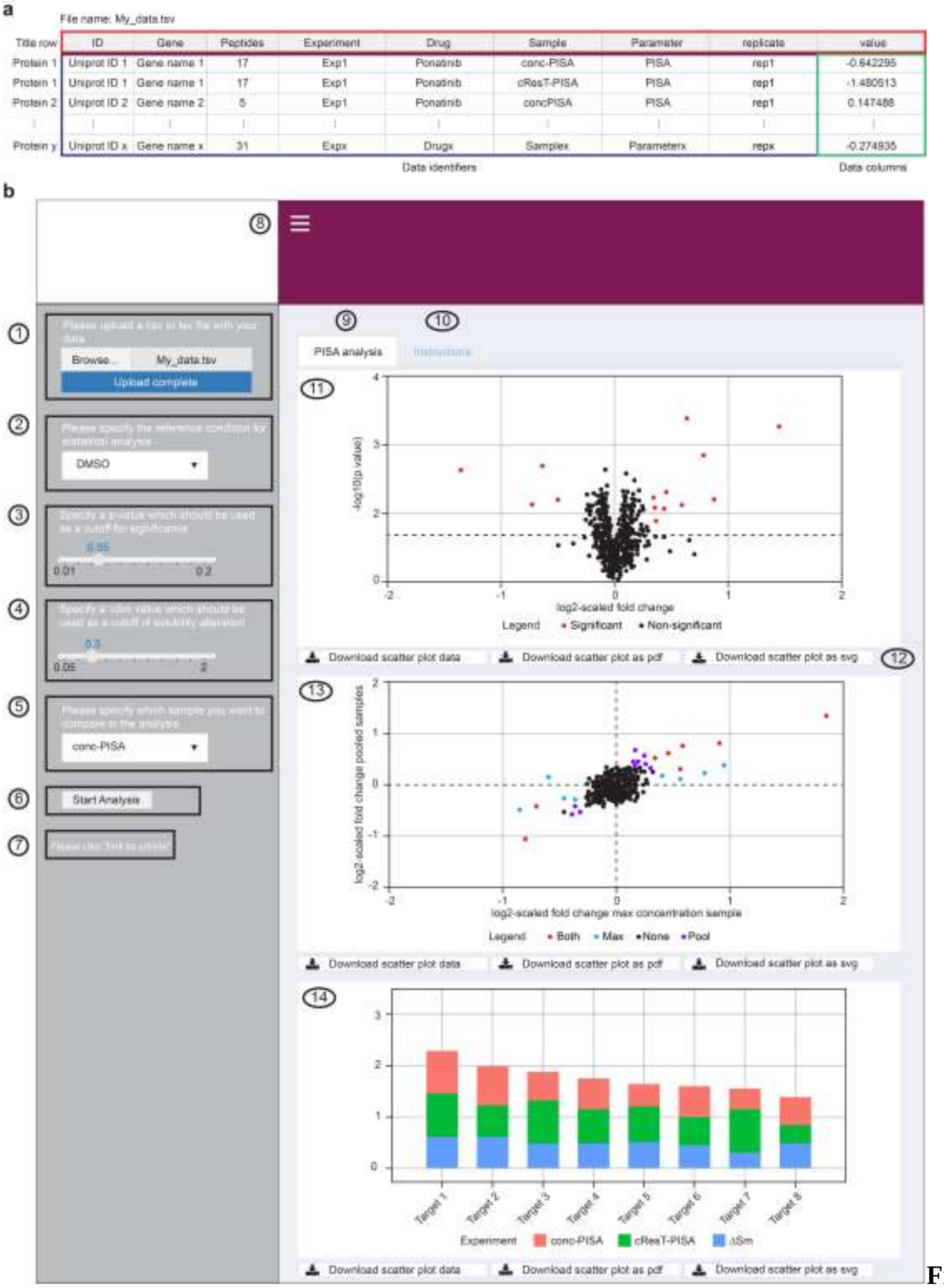

Figure S3. Overview of the PISA-analyser interface. (**a**) Layout of a typical .tsv file containing a proteomics dataset that can be submitted to the interface for analysis. Data columns names should be presented as described here. Any number of conditions and replicates can be included in the .tsv file. Any condition name and replicate name can be included.

(**b**) Step by step description of the web interface.

1) The user can upload own data here.

2) The user needs to specify the reference condition, in most cases the vehicle (DMSO here).

3) The user can specify the p-value cutoff for significance in the analysis.

4) The user can specify the ΔSm cutoff for significance in the analysis.

5) The user needs to specify which sample is to be compared to the reference sample in the analysis.

6) Start button of the analysis.

7) Link to the article.

8) Hide or show the checkbox group.

9) PISA analysis window where the user can see the result of his analysis.

10) Instruction window which displays the figure and caption.

11) Volcano plot of the sample chosen for comparison against the reference condition (from steps 2 and 5) presented as the mean log2 fold change (FC) (x-axis) and -log10 p-value (y-axis). The significant proteins according to the chosen p-value and ΔSm cutoffs are highlighted in red.

12) The plots can be exported as .pdf and .svg, the source data of the plots can also be downloaded as .tsv.

13) Optional plot that will appear only in 2-dimensional experiments (ResT-PISA or conc-PISA). Here two-dimensional plots of target engagement (in this case from maximum concentration of the drug ΔSm) and residence time (in this case from cResT-PISA analysis) presented as the mean log2 FC based on the chosen reference and sample in steps 2 and 5.

14) Optional plot that will appear only in 2-dimensional experiments. Here scoring based on the three dimensions of target engagement (maximum drug concentration), residence time (c-ResT-PISA) and binding affinity (conc-PISA). The score is calculated and is scaled to 1 for each individual parameter as described in the M

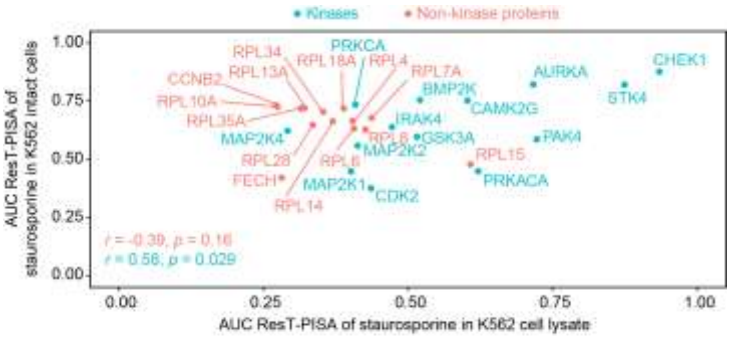

Figure S4. Correlation of ResT-PISA of staurosporine in lysate and in cell. Correlation between the AUC obtained from ResT-PISA of staurosporine in lysate and in cell. Kinase proteins are shown in cyan and non-kinase proteins in red. Pearson’s correlations between the two measures were calculated for the two protein groups.

Extended material and methods

Cell culture. Chronic Myelogenous Leukemia K-562 cell line passage 3-8 (CCL-243™, ATCC) was maintained in non-adherent culture flasks (Sarstedt) in IMDM (Cat. No. L0190, Biowest) supplemented with 10% fetal bovine serum (Cat. No. 10500064, Gibco™).

PISA assay. Each condition was analysed in triplicate. For each replicate, 20 µl of cell suspension were aliquoted into 9 PCR tubes and each was heated for 3 min at 9 different temperatures 48, 49.3, 50.7, 52.2, 53.5, 54.9, 56.3, 57.7 and 59 °C. The samples were then kept for 3 min at RT before snap freezing in liquid nitrogen. After that, aliquots corresponding to each temperature point were combined. For ResT-PISA in cell, one sample designated for protein expression measurement was incubated at 37 °C (n=3 for each condition) and processed alongside the pooled samples. Cells were lysed using repeated freeze/thaw cycles and aliquots were combined as for the lysate experiment. Finally, all samples were transferred to ultracentrifuge tubes, placed into a Ti 42.2 rotor (Beckman-Coulter), and centrifuged at 100 000 x g for 20 min using an Optima XPN-80 Ultracentrifuge (Beckman-Coulter). 70 µl of the supernatant were collected and the same volume of lysis buffer (8 M urea, 20 mM EPPS pH 8.5) was added. The protein concentration was measured using Pierce bicinchoninic acid assay (BCA) protein assay kit (Thermo Fischer Scientific) according to the manufacturer's protocol. The volume of sample was adjusted to the one corresponding to 25 µg of protein and the samples were processed for MS analysis as previously described[26, 27] (see below).

Protein sample preparation for expression proteomics and TMT labeling. For all proteomics experiments, 20 µg of proteins were used in sample preparation. S-S bond reduction was performed using 5 mM DTT at RT for 1 h followed by alkylation using 15 mM IAA at RT in the dark. The reaction was quenched by adding 10 mM of DTT. Then methanol/chloroform precipitation was performed as follows: 3 sample volume of methanol were added, then 1 sample volume of chloroform and 3 volumes of water. Samples were vortexed between each step and then centrifuged at 20 000 x g for 10 min at 4 °C. The aqueous layer was removed, and the protein pellet was rinsed with one sample volume of methanol, vortexed and centrifuged using the same speed as in the previous step. Finally, all the liquid was removed, and the protein pellet was air-dried.

Air-dried protein pellets were resuspended in 8 M urea, 20 mM EPPS pH 8.5. The samples were diluted once by adding 20 mM EPPS pH 8.5 (4 M urea), and lysyl endopeptidase digestion was carried out at a 1:100 ratio (LysC/protein, w/w) overnight at RT. The following day, samples were diluted 4 times (1 M urea) with 20 mM EPPS pH 8.5, then tryptic digestion was performed for 6 h at RT using a 1:100 ratio (Trypsin/protein, w/w). For PISA analysis, a "linker" corresponding to a sample composed of one tenth of each sample pooled together was prepared for normalization purpose. After that, TMT11, TMT16 or TMT18 labeling was performed for 2 h at RT by adding 0.2 mg of reagent dissolved in dry ACN according to manufacturer's instructions. The ACN content in the samples was adjusted to a final concentration of 20%. The reaction was then quenched by adding triethylamine to a final 0.5% concentration. The samples were incubated for 15 min at RT and all temperature points were combined into one pooled sample per replicate. The pooled samples were acidified to pH < 3 using TFA, desalted using Sep Pack (Waters) and vacuum dried overnight using miVac DNA (Genevac).

High pH reversed-phase peptide fractionation. 150 µg of peptides were resuspended in 20 mM NH4OH. Then, samples were off-line high-pH reversed-phase fractionated as described previously[38,39] using an UltimateTM 3000 RSLCnano System (Dionex) equipped with a XBridge Peptide BEH 25 cm column of 2.1 mm internal diameter, packed with 3.5 µm C18 beads having 300 Å pores (Waters). The mobile phase consisted of buffer A (20 mM NH4OH) and buffer B (100% ACN). The gradient started from 1% B to 23.5% in 42 min, then moved to 54% B in 9 min, 63% B in 2 min and stayed at 63% B for 5 min, and finally moved back to 1% B and stayed at 1% B for 7 min. The fractions were collected for 0.7 min each resulting in 96 fractions that were concatenated into 24 fractions (fraction 1 was pooled with fractions 25, 49 and 73, fraction 2 - with fractions 26, 50 and 74 and so on) and dried using miVac DNA (GeneVac, England).

Bioinformatics analysis. All further data processing was performed by a home-written algorithm in R (version 4.1.1). For MS2-based experiments, protein quantification was performed as follows:

1) PSMs mapping to a reverse sequence, known contaminants, with a precursor purity below 0.5 or a PeptideProphet probability below 0.9 were removed.

2) Reporter ion intensities were adjusted to correct for the isotopic impurities of the different TMT reagents by solving a system of linear equations according to manufacturer's instructions. PSMs with corrected TMT intensities below 1000 were removed.

3) If multiple PSMs were detected in the same fraction with the same charge state, the one with the highest purity was retained, while for PSMs with the same purity the one with the highest Hyperscore was selected.

4) Individual protein TMT reporter intensities were calculated as the sum of the individual PSMs.

5) Proteins with less than two unique peptides were removed.

6) To correct for pipetting error, protein TMT reporter intensities were normalized by median centering to the total intensity.

7) Batch effects between different TMT sets were corrected by dividing each protein's TMT intensity by their corresponding linker value.

For the Hyperplexed samples (SILAC-TMT) the protein quantification was performed as followed:

1) PSMs mapping to a reverse sequence, known contaminants, with a precursor purity below 0.5 or a PeptideProphet probability below 0.9 were removed.

2) In each replicate identified PSMs were filtered for matching pairs of light and heavy ones. These pairs were kept together for all further analysis.

3) Reporter ion intensities were adjusted to correct for the isotopic impurities of the different TMT reagents by solving a system of linear equations according to manufacturer's instructions.

4) If multiple PSMs pairs were detected in the same fraction with the same charge state, the one with the highest individual purity was retained, while for PSMs pairs with the same purity the one with the highest individual Hyperscore was selected.

5) Individual protein quantification was calculated based on the sum of the TMT reporter channels of the individual PSMs for light and heavy labeled samples.

6) Proteins with less than two unique light and heavy peptides were removed.

7) To correct for pipetting error, protein intensities were normalized by median centering to the total intensity.

8) Batch effects between different TMT sets were corrected by dividing each protein's TMT intensity by their corresponding light or heavy labeled linker value.

Table S5. LC-MS/MS systems and parameters.

Parameter	Staurosporine Lysate	IAA Lysate	Ponatinib Lysate	Ponatinib PotPot	Staurosporine Cell
**LC system**	Ultimate 3000	Ultimate 3000	Ultimate 3000	Ultimate 3000	Ultimate 3000
**Gradient (min)**	110	210	180	180	135
**Sample multiplexing strategy**	TMT11-plex	TMT10-plex	TMTpro16-plex	TMTpro18-plex	TMTpro16-plex +SILAC
**Mass Spectrometer**	Fusion Lumos	Fusion Lumos	Fusion Lumos	Fusion Lumos	Fusion Lumos
**Scan cycle (s)**	3	3	3	3	2.5
**MS1 resolution**	120`000	120`000	120`000	120`000	120`000
**MS1 scan range (Th)**	400-1600	400-1600	375-1400	375-1400	375-1400
**Injection time (ms)**	50	50	50	50	50
**MS1 AGC**	$4*10^6$	$4*10^6$	$1*10^6$	$1*10^6$	$1*10^6$
**Included charge states**	2-6	2-6	2-5	2-5	2-5
**Mass Difference**	NA	NA	NA	NA	8.0142, 10.0083, 16.0284, 20.0165, 18.0225, 24.0426, 30.0248, 26.0367, 28.0307, Tolerance 10 ppm
**Exclusion duration (s)**	60	60	60	60	45
**MS2 Isolation windows (Th)**	1.6	1.6	0.7	0.7	0.5
**MS2 NCE**	35 (HCD)	35 (HCD)	35 (HCD)	35 (HCD)	35 (CID)

**MS2 resolution**	60`000 (Orbitrap)	50`000 (Orbitrap)	50`000 (Orbitrap)	50`000 (Orbitrap)	Turbo (Ion Trap)
**Injection time (ms)**	118	86	86	86	35
**MS2 AGC**	125`000	125`000	250`000	250`000	25`000
**MS3 Isolation windows (Th)**	NA	NA	NA	NA	1.3
**MS3 NCE**	NA	NA	NA	NA	45 (HCD)
**MS3 resolution**	NA	NA	NA	NA	50`000 (Orbitrap)
**MS3 Injection time (ms)**	NA	NA	NA	NA	86
**MS3 AGC**	NA	NA	NA	NA	250000
